# A high-trans fat, high-carbohydrate, high-cholesterol, high-cholate diet-induced nonalcoholic steatohepatitis mouse model and its hepatic immune response

**DOI:** 10.1186/s12986-023-00749-w

**Published:** 2023-05-27

**Authors:** Qian Zhang, Yue Jin, Xin Xin, Ziming An, Yi-yang Hu, Yajuan Li, Qin Feng

**Affiliations:** 1 Institute of Liver Diseases, Shuguang Hospital Affiliated to Shanghai University of Traditional Chinese Medicine, 528 Zhangheng Road, Pudong District, Shanghai, 201203 China; 2 Institute of Interdisciplinary Integrative Medicine Research, Shanghai University of Traditional Chinese Medicine, 1200 Cailun Road, Pudong New Area, Shanghai, 201203 China; 3 Key Laboratory of Liver and Kidney Diseases, Shanghai University of Traditional Chinese Medicine, Ministry of Education, Shanghai, 201203 China; 4 Shanghai Key Laboratory of Traditional Chinese Clinical Medicine, Shanghai, China

**Keywords:** NASH, HFHCCC mouse model, Model features, Immune response

## Abstract

Non-alcoholic fatty liver disease (NAFLD) is a chronic progressive disease that can progress to non-alcoholic steatohepatitis (NASH). Animal models are important tools for basic NASH research. Immune activation plays a key role in liver inflammation in patients with NASH. We established a high-trans fat, high-carbohydrate, and high-cholesterol, high-cholate diet-induced (HFHCCC) mouse model. C57BL/6 mice were fed a normal or HFHCCC diet for 24 weeks, and the immune response characteristics of this model were evaluated. The proportion of immune cells in mouse liver tissues was detected by immunohistochemistry and flow cytometry, Multiplex bead immunoassay and Luminex technology was used to detecte the expression of cytokines in mouse liver tissues. The results showed that mice treated with HFHCCC diet exhibited remarkably increased hepatic triglycerides (TG) content, and the increase in plasma transaminases resulted in hepatocyte injury. Biochemical results showed that HFHCCC induced elevated hepatic lipids, blood glucose, insulin; marked hepatocyte steatosis, ballooning, inflammation, and fibrosis. The proportion of innate immunity-related cells, including Kupffer cells (KCs), neutrophils, dendritic cells (DCs), natural killer T cells (NKT), and adaptive immunity-related CD3+ T cells increased; interleukin-1α (IL-1α), IL-1β, IL-2, IL-6, IL-9, and chemokines, including CCL2, CCL3, and macrophage colony stimulating factor (G-CSF) increased. The constructed model closely approximated the characteristics of human NASH and evaluation of its immune response signature, showed that the innate immune response was more pronounced than adaptive immunity. Its use as an experimental tool for understanding innate immune responses in NASH is recommended.

**Supplementary Information:**

The online version contains supplementary material available at 10.1186/s12986-023-00749-w.

## Introduction

Nonalcoholic fatty liver disease (NAFLD) is gradually becoming one of the most frequent liver diseases worldwide. NAFLD includes a broad spectrum of diseases ranging from nonalcoholic fatty liver to nonalcoholic steatohepatitis (NASH), cirrhosis, and hepatocellular carcinoma [1]. In recent years, the incidence of NASH-related mortality has rapidly increased. Research on the pathogenesis of NASH and drug development has been conducted like a raging fire. For this process, a suitable animal model is required. Animal models that are as similar as possible to human NASH disease characteristics are needed for pathogenesis and drug development.

Excessive energy intake and insufficient energy expenditure are the main factors leading to the occurrence of fatty liver. An animal model based on diet-induced excess nutrition is one of the most common NASH models. A high-fat diet is currently the most classic and commonly used NAFLD model. Commonly used fats mainly include saturated fats, such as lard and tallow, and unsaturated fats, such as soybean oil. In addition to high fat, high sugar is the main factor that induces the occurrence and development of fatty liver. Carbohydrates, such as fructose and sucrose, are not only stimulate de novo lipogenesis but also induce hepatic oxidative stress and inflammation [2]. Compared with the simple high-fat diet, high fat combined with high sugar intake requires a relatively short time for modeling, and the degree of disease is relatively severe [3]. Although conventional high-fat and high-sugar diets can induce pathological features of NASH, the fibrotic features of the model are not obvious. Researchers have attempted to add other ingredients to accelerate disease and fibrosis progression.

Trans fat is an unsaturated fat that can lead to increased expression of lipogenic genes in the liver and enhanced phagocytosis of KCs [4], which can exacerbate steatosis, inflammation, and fibrosis [5]. Cholesterol plays an important role in the progression of NAFLD to NASH, which has been shown to exacerbate progression [6, 7]. Adding bile salts to high-cholesterol feeds can enhance cholesterol absorption and inhibit cholesterol clearance, which is conducive to the deposition of fat in the body. Many studies have confirmed the role of high trans fatty acids, high sugars, and high cholesterol in NASH models, but few studies have involved the simultaneous use of high-trans fat, high-carbohydrate, high-cholesterol, high-cholate (HFHCCC) models to induce NASH. Therefore, in this study, we aimed to explore an animal model that is close to the histological characteristics of patients with NASH and simultaneously shows more prominent NASH characteristics. High fat diets containing trans fats with increased cholesterol and bile salts were used in combination with high-fructose drinking water for 24 weeks to induce a mouse model of NASH.

Evidence is mounting that the immune response plays an important role in the progression of NASH [8]. Glycolipid metabolism disorders caused by high fat and sugar levels can induce liver immune disorders and inflammation. Immune disorders aggravate metabolism disorders, forming a vicious circle [9]. Immune responses include innate and adaptive immunity, both of which are involved in NASH-related inflammation. For the purpose of revealing the formation mechanism of the model better, especially the immune mechanism, we conducted a comprehensive and systematic analysis of the immunological characteristics of the model as a whole to provide a basis for clarifying the immune mechanism of the model.

## Materials and methods

Five-week-old male C57BL/6 mice, purchased from Shanghai Slack Laboratory Animal Center (Shanghai, China), were group-housed in the Animal Center of Shanghai University of Traditional Chinese Medicine. Mice were maintained in a 12:12-h light–dark cycle schedule. Mice were randomly divided into either normal diet (10% fat, Trophic Animal Feed High-tech Co., Ltd, China, TP23301S) and normal water or high-fat, high-carbohydrate, high-cholesterol, and high cholate diet (HFHCCC) (45% of the feed energy was derived from fat, trophic Animal Feed High-tech Co., Ltd, China, TP23302S), and drinking water enriched with high fructose. Sugar water with a concentration of 42 g/L was made from drinking water at a ratio of 55% fructose and 45% sucrose by weight. Bile salt is 0.5% sodium cholate. The composition of each diet is presented in Supplementary Tables 1 and 2. The animals were provided ad libitum access to the diet for 24 weeks. All animals were sacrificed for tissue collection at the end of the 24th week. All animal procedures were performed in line with the National Institutes of Health Guidelines for Laboratory Animals and were approved by the Animal Ethics Committee of Shanghai University of Chinese Medicine (Additonal files 1, 2).

### Biochemical assays

Animals were sacrificed after 24 weeks, and serum was obtained by taking the eyeballs after an overnight fast. Serum glucose (GLU) was measured with a glucose quantification kits according to the manufacturer’s instructions (Nanjing Jian Cheng Bioengineering Institute, Nanjing, China). Serum insulin levels were determined using an ultra-sensitive mouse insulin ELISA kit (18APUMI482A). The hepatic liver triglyceride (TG) content was obtained from triglyceride kit according to the manufacturer's instructions (Dongou Bioengineering, Zhejiang, China) at 24 weeks. Total cholesterol (TC), low-density lipoprotein cholesterol (LDL-C), and high-density lipoprotein cholesterol (HDL-C) levels were detected using a TOSHIBA TBA-40FR Automatic Analyzer (Hitachi, Limited, Tokyo, Japan).

### Histology

Liver tissues were collected from the mice after 24 weeks, fixed in 10% formalin, paraffin embedded, and sectioned into 4 μm slices. Tissue sections were differentiated with 1% hydrochloric acid alcohol for 5–10 s and then stained with hematoxylin and eosin (H&E). Sirius Red was used to visualize the degree of fibrosis and collagen deposition. Analysis was performed using Sirius red-stained area aperture image scope-pathology slide viewing software. Liver fibrosis stage was determined using a previously published fibrosis staging system [10].

### Immunochemistry

Liver tissues were collected from the mice after 24 weeks and fixed in 10% formalin. Immunostaining was performed using 7 μm formalin-fixed, paraffin-embedded sections. Deparaffinized tissue sections were subjected to antigen retrieval. Col-1 (collagen type-1) and sodium citrate buffer were used for antigen retrieval of α-SMA (α-smooth muscle actin) in liver tissue sections. The repair method for MPO was the high-pressure repair method using sodium citrate buffer. The method used for F4-80 was the EDTA high-pressure repair method. Endogenous peroxidase activity was quenched. Mouse monoclonal anti-PAR antibody (Trevigen, Gaithersburg, MD) was used at a 1:300 dilution overnight at 4 °C, and then mixed with goat anti-mouse F4/80 (KCs marker) (1:100, Abcam, ab111101), MPO (myeloperoxidase, neutrophil marker) (1:100, Abcam, 22225-1-AP), COL-1 (1:100, Abcam, ab34710), α-SMA (1:100, Abcam, ab5694), and biotinylated goat anti-rabbit IgG (Biotech Well, WH1057-2), followed by signal amplification with streptavidin and final counterstaining with hematoxylin.

### Flow cytometry

After anesthesia of mice, the inferior vena cava was taken blood, the inferior vena cava was cut after the blood collection was completed, the 200-purpose stainless steel cell sieve was placed on the petri dish, the liver was removed and placed on the cell sieve, immersed in a petri dish containing 0.2% BSA-PBS, the liver was milled with the tail of the 1 ml syringe suction cylinder, and the fully ground cell suspension was fixed to 15 ml, 650 rpm, 1 min, discarded supernatant, retained cell pellet, and 0.2% was added again BSA-PBS centrifuged again and discarded the supernatant, left the cell pellet, and added a little PBS to mix the pelleted cells to prepare a single-cell suspension. Single liver cell suspensions were centrifuged at 1500 rpm, the suspension was divided into three layers, and the milky white membrane layer in the middle was the lymphocytes, then the lymphocytes were separated and resuspended in 70% Percoll. Cell suspension was centrifuged at 2000 rpm for 5 min at room temperature, and then 1*10^6 cells from each sample were drawn and suspended in 29.5 μl 0.2% BSA-PBS buffer, blocked with anti-mouse CD16/32 (Becton, Dickinson and company, 553141), and incubated at 4 °C for 15 min. Cells were stained with FITC-CD3 (Becton, Dickinson and Company, 553061), PerCP-CD4 (Becton, Dickinson and Company, 553052), PE-Cy7-CD8 (Becton, Dickinson and Company, 552877), APC-Cy7-CD19 (Becton, Dickinson and Company, 557655), APC-NK1.1 (Becton, Dickinson and Company, 550627), and PE-CF594-CD11C (5552454; Becton, Dickinson and Company, USA). Cells were acquired on a Beckman Flow Cytometer (BECKMAN, COULTER, DEFLEX), and the data were analyzed using FlowJo software version 7.6 (TreeStar, Ashland, OR).

### Detection of cytokine protein levels in liver tissue

Multiplex bead immunoassay and Luminex technology (Bio-PlexPro Mouse Cytokine7-plex panel, Bio-Rad) were used to determine the cytokine levels in the mouse liver. The following 23 cytokines: interleukin-like pro-inflammatory cytokines including interleukin-1α(IL-1α), interleukin-1β(IL-1β), interleukin-2 (IL-2), interleukin-6 (IL-6), interleukin-9 (IL-9), interleukin-12 (P40) (IL-12 (P40)), interleukin-12 (P70) (IL-12 (P70)), interleukin-17A (IL-17A), Interleukin-like anti-inflammatory cytokines including interleukin-4 (IL-4), interleukin-5 (IL-5), interleukin-10 (IL10), interleukin-13 (IL13); tumor necrosis factor (TNF-α), γ-Interferon (IFN-γ); chemokine including c–c motif chemokine 2 (CCL2), c–c motif chemokine 3 (CCL3), c–c motif chemokine 4 (CCL4), c–c motif chemokine 5 (CCL5), c–c motif chemokine 11 (CCL11), C-X-C motif chemokine 2 (CXCL2); colony stimulating factor including granulocyte–macrophage colony stimulating factor (GM-CSF), macrophage colony stimulating factor (G-CSF) and pluripotent colony stimulating factor (IL-3) could simultaneously be analyzed by an immunoassay kit. After liver tissue trituration and homogenization, all samples were diluted 1:2 with lysis buffer (Bio-Rad Laboratories, Hercules, CA, USA). After sonication, Supernatant Protein samples were diluted 1:3 with Bio-Plex Sample Diluent and used for multiplexing at protein concentrations of approximately 3–4 mg/ml. Finally, the beads were loaded onto a Bio-Plex 200 system (Bio-Rad) for analysis.

### Statistical analysis

Statistical analysis was performed using GraphPad Prism version 8.0 GraphPad Software, La Jolla, CA). Data are expressed as mean ± standard error of measurement (SEM). Data were analyzed using test-test for comparisons of two groups. For comparison of more than two groups, ANOVA multiple comparison test was used, and *p* < 0.05 was regarded statistically significant.

## Results

### Total calories, body weight, liver weight, liver body ratio, blood glucose, lipids and insulin

After 24 weeks of HFHCCC administration, the total calories consumed did not differ between the control and HFHCCC groups (Table 1), and the body weight in the HFHCCC group, was lower than that in the control group (*P* < 0.01,Table 1). By contrast, HFHCCC exposure increased liver weight and liver body ratio (*P* < 0.01, Table 1). Notably, CIH exposure leads to abnormal glucose metabolism in the liver. The fasting blood-glucose (FBG) was higher in HFHCCC group than in control group (*P* < 0.01, Table 1). The fasting insulin level was also higher in HFHCCC group compared to control group (*P* < 0.05, Table 1). In terms of lipid metabolism, there were no significant differences in the serum TG and TC levels between the two groups (Table 1). LDL-C in liver tissue was significantly increased in the HFHCCC group than in control group (*P* < 0.05, Table 1) and there was a trend towards decreased HDL-C in the HFHCCC group (Table 1).

**Table 1:** Total calories, body weight, liver weight, liver body ratio and blood glucose, lipids and insulin of mice treated with HFHCCC Diet compared with the control group

	CON (n = 8)	HFHCCC (n = 8)
Energy intake (kcal/d)	7.20 ± 2.47	7.15 ± 1.94
Body weight (g)	32.39 ± 2.79	24.57 ± 9.40**
Liver weight (g)	1.07 ± 0.04	1.65 ± 0.20**
Liver body ratio	0.03 ± 0.00	0.06 ± 0.00**
TC (mmol/l)	2.88 ± 0.20	2.98 ± 0.28
TG{mmol/l)	0.5 ± 0.19	0.33 ± 0.04
LDL-C {mmol/l)	0.39 ± 0.04	0.55 ± 0.14**
HDL-C (mmol/l)	1.2 ± 0.09	0.93 ± 0.29
FBG (mmol/l)	5.24 ± 0.74	7.00 ± 1.32**
FINS (ng/ml)	0.68 ± 0.13	1.05 ± 0.42*
HOME-IR	0.19 ± 0.05	0.41 ± 0.24*

CON: control diet; HFHCCC: high-fat/carbohydrate/cholesterol/choline diet *P < 0.05, **P < 0.01

### HFHCCC Induced hepatic steatosis, inflammation and fibrosis

**Fig. 1:**
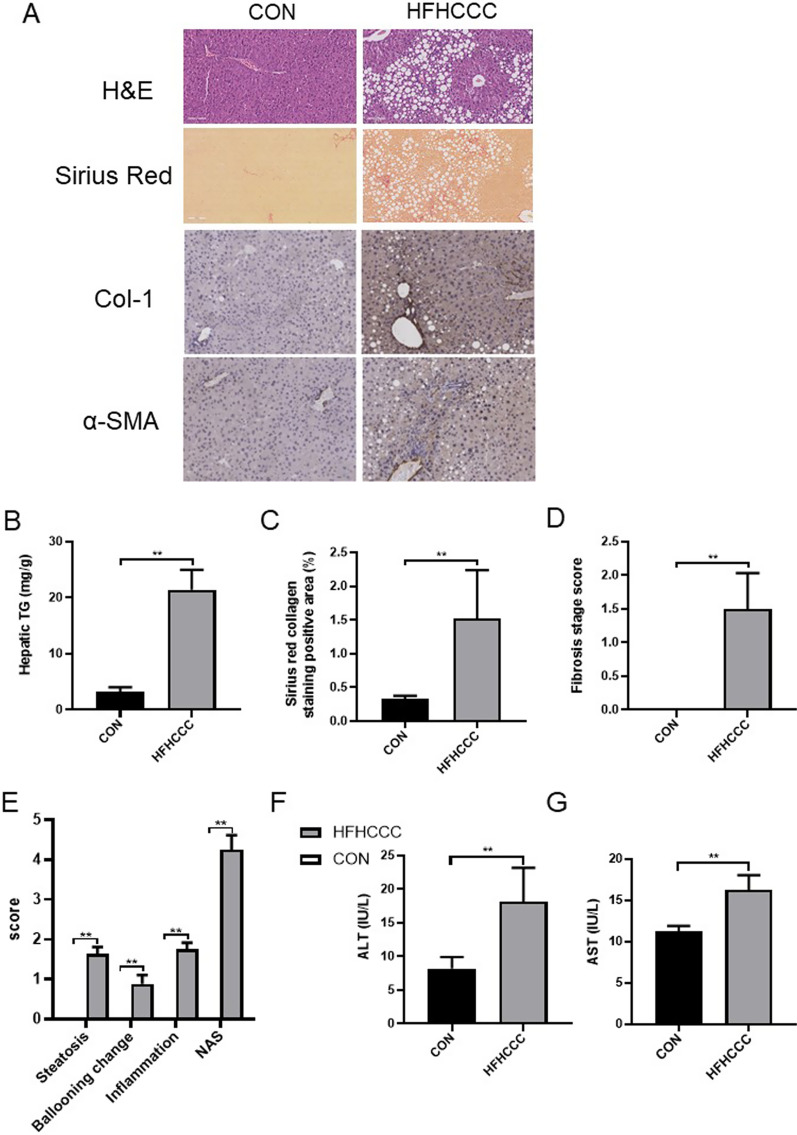
HFHCCC Induced hepatic steatosis, inflammation and fibrosis in mice. **A** H&E (× 40 magnification) and SR staining (× 40 magnification) on liver sections, col-1, α-SMA immunohistochemical staining (× 200 magnification). **B** Hepatic TG content. **C** Sirius red collagen staining possitive area. **D** Fibrosis stage score. **E** NAS score. **F** Serum ALT activity. **G** Serum AST activity  of the mice in each group. *P < 0.05, **P < 0.01

Hematoxylin and eosin (H&E) staining showed substantial liver steatosis with inflammatory changes in the HFHCCC group, and micro-and macrovesicular steatosis were clearly visible after 24 weeks. Almost all hepatocytes were enlarged owing to excessive lipid droplet deposition, and inflammatory cell infiltration was observed in the liver tissues. Sirius red staining showed remarkable liver fibrosis in the HFHCCC group (Fig. 1a), and fibrosis was widespread in most portal areas. Immunohistochemical (IHC) staining of liver tissue showed that the protein expression of COL-1 and α-SMA was remarkably increased in the HFHCCC group (Fig. 1a). Moreover, the collagen-positive staining area in the HFHCCC group was significantly increased (*P* < 0.01, Fig. 1c), and the fibrosis score was approximately 1.5 points (*P* < 0.01 vs control, Fig. 1d). The steatosis, ballooning, inflammation, and NAFLD activity (NAS) scores in the HFHCCC group were significantly higher than those in the control group (*P* < 0.01, Fig. 1e). Liver tissue TG levels were higher in the HFHCCC group than in the control group (Fig. 1b). In addition to histological assessment, we also examined markers of liver damage associated with NASH. The serum activity of ALT and AST  also showed significant increase in HFHCCC treat mice (*P* < 0.01, Fig. 1f, g).

### HFHCCC induced changes in innate immunity

**Fig. 2:**
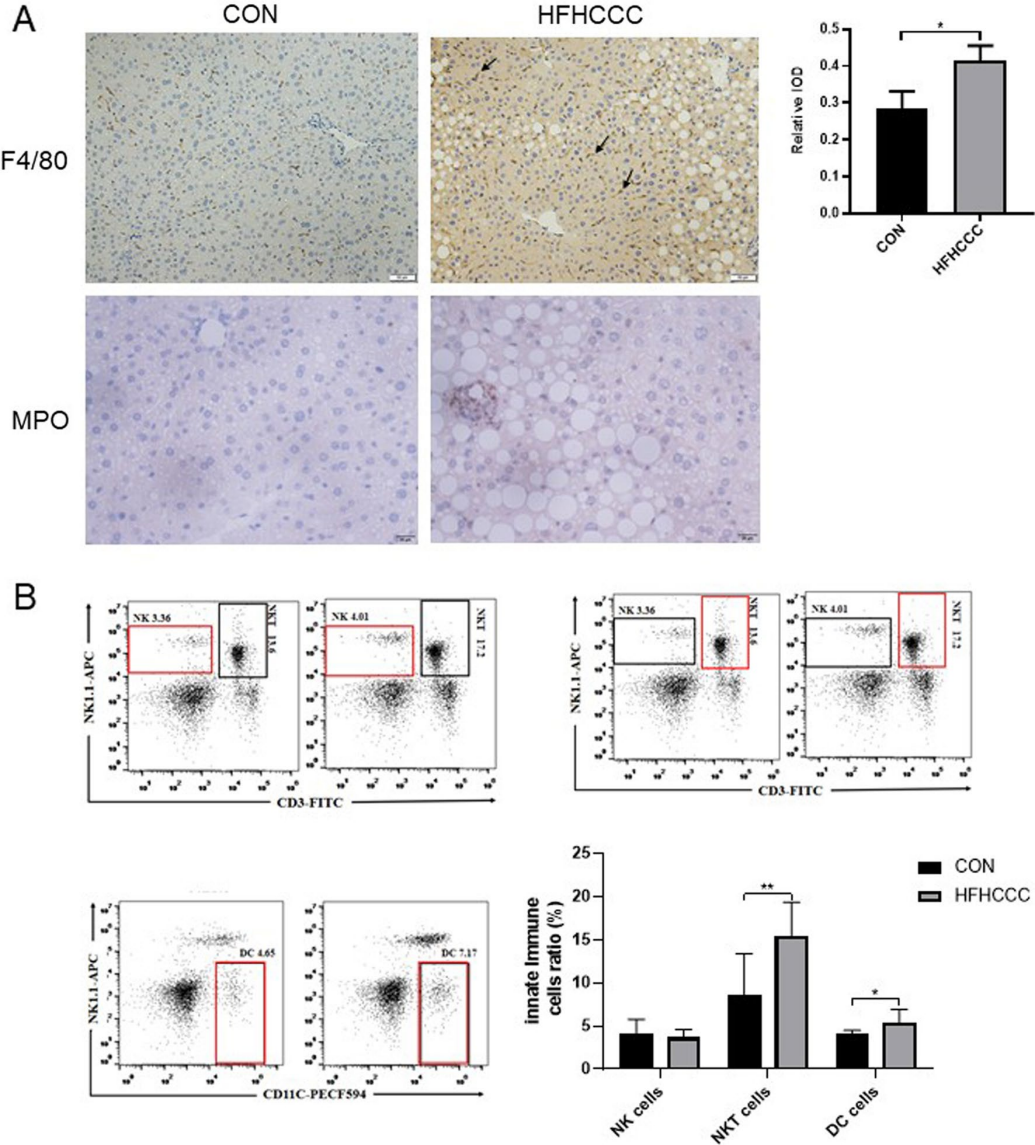
HFHCCC Induced changes in innate immune state in mice. **A** Macrophage F4/80 and Neutrophil MPO immunohistochemical staining results on liver tissue. **B** The proportion of NKT cells, NKT cells, DC cells in mouse liver. *P < 0.05, **P < 0.01

Liver tissue IHC staining showed more positive F4/80 staining in the perivenous hepatic sinusoids in the HFHCCC group than in the control group (Fig. 2a), and there was a large number of neutrophils around the inflammatory foci (Fig. 2a). There was no significant difference in the proportion of natural killer (NK cells) in the liver tissue between the two groups by flow cytometry assays, whereas the proportion of DCs and NKT cells were striking increased in the HFHCCC group compared to those in control group (*P* < 0.05, Fig. 2b).

### HFHCCC induced changes in adaptive immunity

**Fig. 3:**
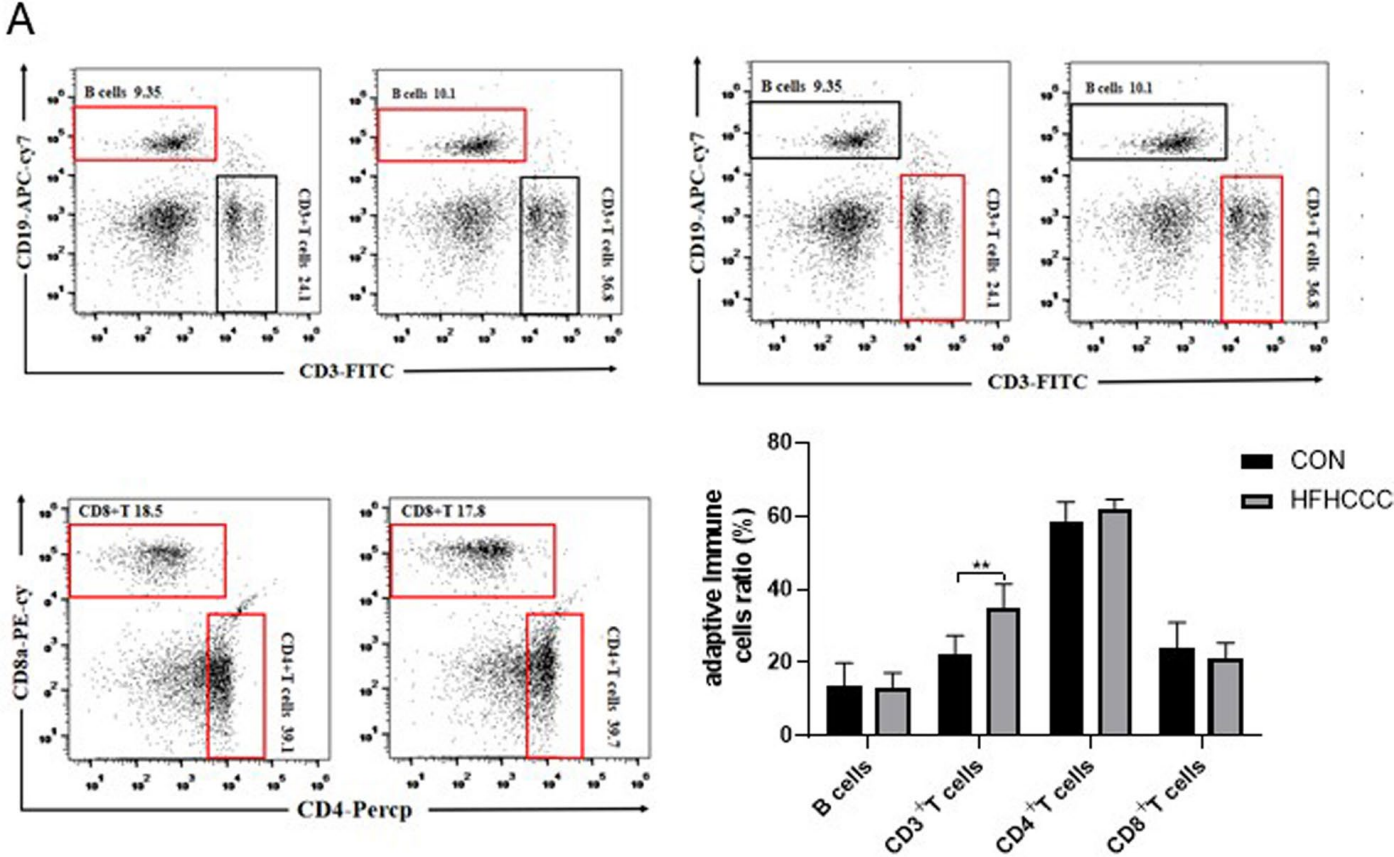
HFHCCC Induced changes in adaptive immune state in mice (**A**). The proportion of B cells, CD3 + T cells, CD4 + T cells, CD8 + T cells. *P < 0.05, **P < 0.01

The liver immune cell flow cytometry assay showed that CD3+ T cells were upregulated, while no significant changes in the proportion of B cells, CD4+ T cells, and CD8+ T cells between the two groups were observed (*P* < 0.01, Fig. 3a).

### HFHCCC induced changes in hepatic cytokine levels

**Fig. 4:**
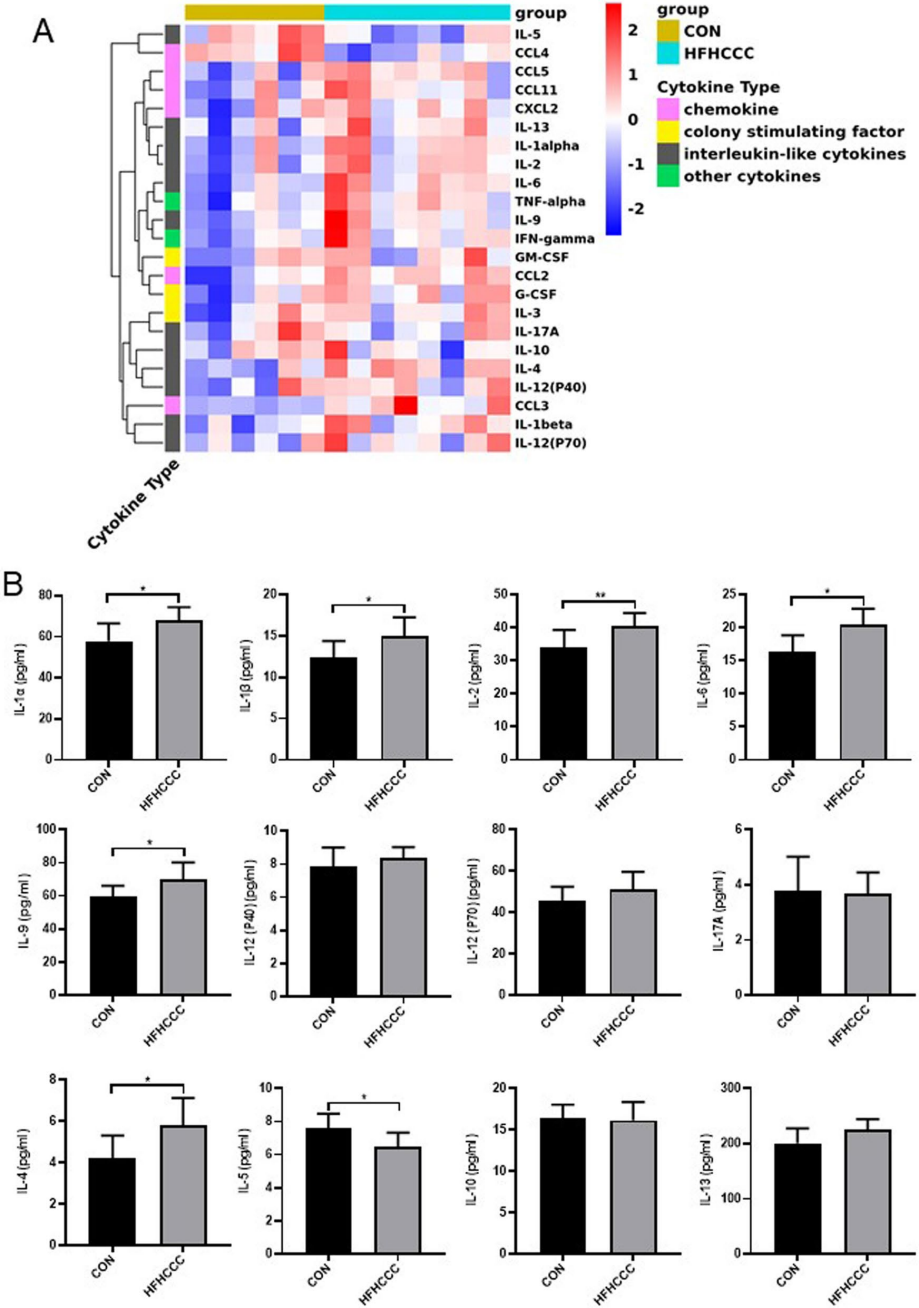

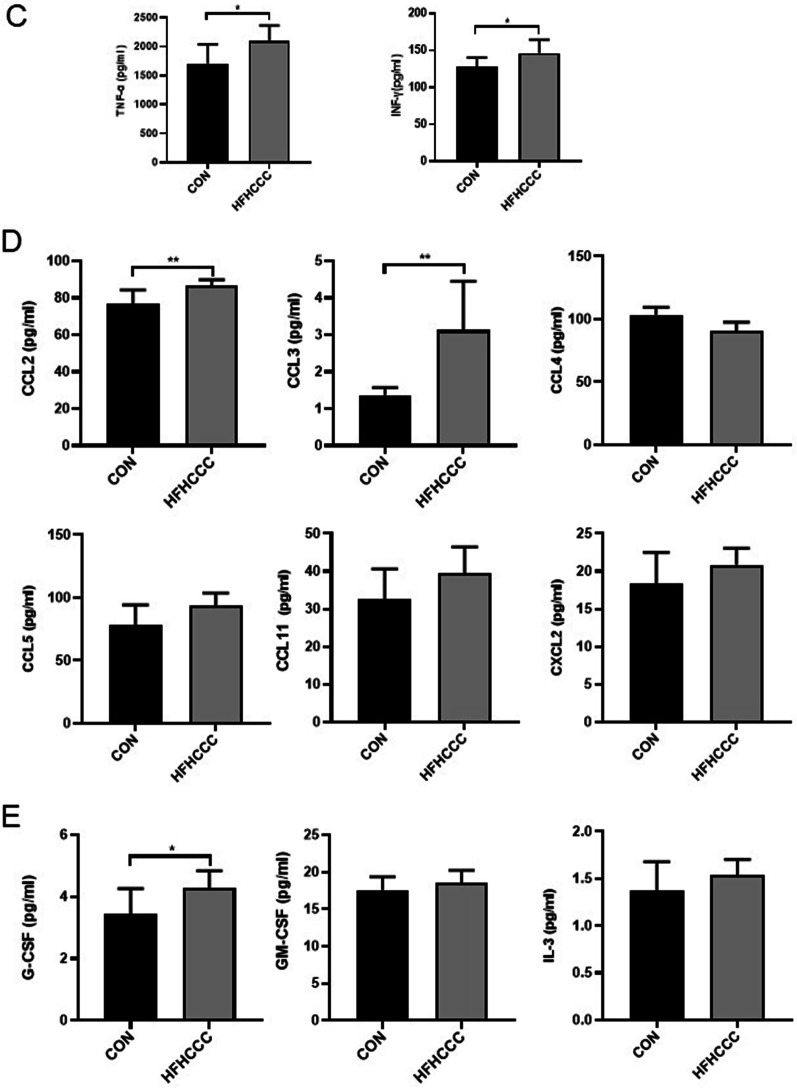
HFHCCC Induced changes in the proportion of hepatic cytokine levels. **A** Heatmap of serum inflammatory cytokines. Rows represent cytokine species and columns represent sample groups. **B** Interleukin-like pro-inflammatory cytokines IL-1α, IL-1β, IL-2, IL-6, IL-9, IL-12 (P40), IL12 (P70), IL-17A, Interleukin-like anti-inflammatory cytokines IL-4, IL-5, IL-10, IL-13 protein levels.*P < 0.05, **P < 0.01. **C** The protein levels of tumor necrosis factor TNF-α and interferon-like inflammatory factor IFN-γ. **D** The protein levels of chemokines CCL2, CCL3, CCL4, CCL5, CCL11, CXCL2. **E** The protein levels of colony stimulating factor GM-CSF,G-CSF,IL-3. *P < 0.05, **P < 0.01

Figure 4a showed a heatmap of the 23 cytokines in the liver tissue. Compared to control group, the cytokines in the HFHCCC treatment group showed an overall increasing trend. We further analyzed the changes in the levels of these 23 cytokines. The protein expression of interleukin-like pro-inflammatory cytokines IL-1α, IL-1β, IL-6, and IL-9 was strikingly upregulated in the HFHCCC group (*P* < 0.01, vs. control, Fig. 4b), and the protein expression of IL-2 was observably upregulated in the HFHCCC group (*P* < 0.05, vs. control, Fig. 4b), whereas the expression of interleukin-like anti-inflammatory cytokine IL-5 was downregulated in the HFHCCC group (*P* < 0.05, Fig. 4b). There were no significant differences in IL-12 (P40), IL-12 (P70), IL-17A, and IL-13 levels between the two groups (Fig. 4b). HFHCCC-diet treated mice exhibited elevated TNF-α and IFN-γ levels relative to mice on control diet (*P* < 0.05; Fig. 4c). The protein expression of chemokines CCL2 and CCL3 was significantly increased in the HFHCCC group (*P* < 0.01 vs control, Fig. 4d), whereas no notable changes in CCL4, CXCL5, CXCL2, and CCL11 were found between the two groups (Fig. 4d). Regarding colony-stimulating factors, the HFHCCC group exhibited significantly increased expression of hepatic G-CSF (*P* < 0.05, vs. control, Fig. 4e). No significant differences in hepatic GM-CSF and IL-3 levels were observed between the two groups (Fig. 4e).

## Discussion

Our study showed that NASH mice induced by a high-fat, high-sugar diet with trans-fat as the main fat for 24 weeks with an increased ratio of cholesterol and bile salts exhibited advanced liver inflammation, hepatic steatosis, and fibrosis. After the HFHCCC diet model, significant changes were observed in the levels of innate immune cells and their released cytokines in the liver of mice, while the related changes in adaptive immune cells were not obvious. This model is more inclined to trigger an innate immune response than an adaptive immune response.

After 24 weeks of HFHCCC diet induction, the content of TG, as the main indicator of liver fat content, in hepatic mice had significantly elevated. Mice showed significant hyperglycemia and insulin resistance. Pathologically, NASH in mice is characterized by hepatic steatosis, infiltration of neutrophils and macrophages. The stage of liver fibrosis in mice is mostly F1-2. The primary clinical manifestation of NASH includes dyslipidemia, steatosis, and liver cell damage, inflammation, and varying degrees of fibrosis [11]. In this study, the main features of the HFHCCC-induced NASH model were very consistent with the main clinicopathological features of NASH patients.

Theoretically, the "ideal" NASH model should reflect the full spectrum of biochemistry and histology of clinical human liver disease, as well as the characteristics of the associated metabolic syndrome, and should not be too long (e.g., more than one year) [12]. At present, commonly used NASH models include methionine choline deficiency diet (MCD) model, carbon tetrachloride diet (CCL4) model, high-fat diet (HFD) model, high-fat and high-sugar diet (HFHC) model, etc. Although a MCD or CCL4 can induce fibrosis, but it cause weight loss that does not exhibit pathological characteristics similar to that of humans, and lack of insulin resistance or promote fibrosis without hepatic steatosis. Diet-induced obesity models are more similar to the physiology of human NASH patients. However, significant liver fibrosis couldn’t be developed by a high-fat diet alone in most rodents [13]. C57BL/6J mice fed a traditional high-fat diet (60% of energy comes from fat) showed obesity and disorders of glycolipid metabolism after 10 weeks, steatosis and inflammation at 16 weeks, and took 50 weeks to induce mild fibrosis [14]. After 30 weeks of feeding C57BL/6 mice a high-fat and high-sugar diet, they showed NASH characteristics such as balloon-like change, glucose and lipid metabolism disorders, liver damage, and inflammation, but the degree of fibrosis was very mild [7]. Compared with these conventional high-fat diet, high-fat and high-sugar diet models, models with added cholesterol and bile salts can better induce disease characteristics similar to those in humans. Mels et al. [15] found that C57BL/6J mice modeled with a high-trans-fat, high-sucrose and high-cholesterol diet not only developed steteaosis, lobular inflammation, hepatocyte balloon-like transformation, but also showed fibrosis. Tu et al. [16] induced NASH with high-fat and high-cholesterol bile salt diet modeling, in which 37.1% of the energy came from fat, the cholesterol content was 1.25%, and the sodium cholate content was 0.5%, the pathological results showed that the mice showed steatosis and inflammation after the diet modeling, but the degree of fibrosis was very low, our study increased the cholesterol content (2%) and increased high-sugar drinking water based on the research of Lan N. Tu et al. further accelerating the progress of NASH. More pronounced disorders of glycolipid metabolism were induced, and fibrosis occurred. In summary, the HFHCCC diet was more capable of replicating NASH models similar to human metabolic and histological characteristics than other methods-induced NASH models.

However, it was worth noting that although the HFHCCC diet-treated mice showed significantly increased liver weight and liver body ratio compared to control diet-treated mice, yet mice in the HFHCCC group exhibited significant decreased body weight by approximately 9% at 24 weeks compared to that in the control group. A possible reason is that sodium cholate can participate in the metabolism of bile acids and promote the metabolism and absorption of cholesterol, activate G protein-coupled receptor 5, induce the activity of type 2 deiodinase, promote the conversion of tetraiodothyronine to triiodothyronine, and finally promote the energy expenditure, resulting in weight loss [17]. In conclusion, this model could simulate the entire disease course of human NASH patients, while exhibiting the characteristics of NASH fibrosis, showing characteristics of patients with NASH, especially non-obese patients with NASH liver fibrosis.

More and more evidences show the role of immune response in the pathogenesis of NASH. The innate immune cells of liver include Kupffer cells (KCs), neutrophils, dendritic cells (DC) and natural killer cells (NK). In order to further analyze the immune disorder of the model, we detected the expression of immune cells and related inflammatory factors in liver tissue. The results showed that HFHCCC model induced the activation of KCs, neutrophils, DCs and other innate immune cells in the liver of mice, accompanied by increased release of cytokines; However, most of the adaptive immune-related cells in the mouse liver did not change, and the cytokine released by the adaptive immune cells did not change significantly. The results suggested that HFHCCC diet was more likely to activate the innate immunity of mice.

Clinical studies had found that immune cells were activated at all stages of human NAFLD, including T cells, B cells, macrophages and neutrophils [18]. The recruitment of KCs and the expression of pro-inflammatory cytokines such as CCL2, TNF and IL-1βcould be observed in the liver tissue of patients with NASH [19]. After being activated, KCs could secrete pro-inflammatory cytokines such as CXCL2, CXCL1 and IL-8, promoted the recruitment and differentiation of other immune cells, such as neutrophils and dendritic cells, to regulate the immune response under metabolic stress. Some studies found that NASH tended to activate innate immunity before fibrosis F0-1, and adaptive immune response during fibrosis F2-4 [20], macrophages were the first immune cells detected in patients with NASH, and could be detected at the stage of steatosis. However, the increase of CD3+ T cells, CD4+ T cells, and CD8+ T cells in the liver or peripheral blood could not be observed until NASH fibrosis F2-4. In this study, HFHCCC diet was used to induce NASH liver fibrosis, with the stage of F1-2, and KCs cells were observed in the liver tissue of mice at 24 weeks The significant recruitment of neutrophils and the recruitment and release of pro-inflammatory cytokines, chemokines and colony-stimulating factors, but CD4+ T cells, CD8+ T and other adaptive immune-related cells and cytokines did not change significantly before and after the HFHCCC diet modeling, which is also basically consistent with the results of human research.

Many NASH models have been used in the related research of NASH immune response, including MCD diet-induced NASH mouse model [21], HFD diet-induced NASH mouse model [22], HFHC diet-induced NASH mouse model [23], ob/ob mouse model [24], etc. Studies have found that MCD diet can induce both innate and adaptive immunity. However, the current studies on the immune response of NASH induced by MCD diet mostly focus on KCs, NKT cells, Inflammasome and other innate immune responses. After using NASH induced by MCD diet, it was found that KCs [22],NKT cells [21], DC cells [25] and NK cells [26] were significantly activated, and CD4T lymphocytes were significantly recruited [27], cytokines such as IFN-γ, IL1α, IL1β, IL12 (p40), GM-CSF, and CCL3 secreted by these cells are also higher. Similarly, the liver tissue of NASH mice induced by HFD also showed significant activation of KCs [22], neutrophil infiltration [28] and large increase of DC cells [29], increased release of pro-inflammatory cytokines such as TNF-α, IL-6, MCP-1, etc. released by innate immune cells. Kind et al. [30]. found that the activation of innate immune cells such as NK cells and DC cells appeared in the liver tissue and adipose tissue of mice for 15 weeks, while there were no significant changes in the effector T cell subsets. We observed macrophage and neutrophil infiltration and TNF in the liver tissue of mice 24 weeks after modeling with HFHCCC diet and found macrophage and neutrophil infiltration and release of other related inflammatory factors such as TNF-α, IL-6, IL-1α, IL-1β and in mouse liver tissues 24 weeks after modeling with HFHCC diet. Higher levels of NKT cells and DC cells were detected, but no changes in adaptive immune-related cells and cytokines such as CD4+ T cells and CD8+ T cells were found. To some extent, it is proved that the immune characteristics of NASH model induced by MCD diet and HFD diet are similar to those of our study.

It is worth noting that there are some immune cells whose role in NASH is complex and controversial. Different studies on NKT cells have shown different conclusions. Zheng et al. [31] constructed two models, high-fat and high-sugar diet model and MCD model, to induce fibrosis in NASH mice, and found that the liver NKT cells of mice fed a high-fat and high-sugar diet were significantly less than those in the normal diet group, while the abundance of liver NKT cells in mice fed the MCD diet did not change significantly compared with the normal diet group. A human immune-related study found NKT cell aggregation in the fibrous septum in patients with NASH stage 3–4 liver fibrosis [32]. Our findings show significant recruitment of NKT cells after dietary induction of HFHCC, which is closer to the human findings. The results of different studies vary, which may be related to the different stages of disease development caused by different modeling methods.

This study had some potential limitations. Inconsistent with the disease characteristics of most patients with NASH, the weight of the model mice was lower than that of the normal group, which was mainly related to the involvement of bile salts in bile acid metabolism affecting energy metabolism; on the other hand, this model also provided a reference for the study of non-obese NASH. In addition, due to the limitation of the number of experimental channels for flow analysis, the study only used immunohistochemistry for the analysis of liver macrophages, and lacked the results of flow analysis; in terms of adaptive immunity, Th1, Th2, Th17, and Treg have not been further detected and analyzed, which is also the direction for further research in the later stage.

In conclusion, we established an HFHCCC diet-induced NASH model that is stable and reproducible, showing the emergence of NASH pathological features such as inflammation, steatosis, and fibrosis. This model is likely to trigger innate immunity. This could serve as a suitable experimental model for drug testing and for understanding the pathogenesis of innate immunity in NASH.

## Supplementary Information

**Additional file 1**. Composition of HFHCC diet and control diet.

**Additional file 2**. Energy composition of HFHCC diet and control diet.

## Data Availability

The datasets used and/or analyzed during the current study are available from the corresponding author upon request.

## Abbreviations

NAFLD: Nonalcoholic fatty liver disease
NASH: Nonalcoholic steatohepatitis
MCD: Methionine and Choline Deficient L-Amino Acid Diet
HFD: High-fat diet
HFHC: High-fat, high-cholesterol diet
HFHCCC: High-trans fat, high-carbohydrate, high-cholesterol and high-cholate diet
TG: Triglyceride
TC: Total cholesterol
LDL-C: Low-density lipoprotein cholesterol
HDL-C: High-density lipoprotein cholesterol FBG: Fasting blood glucose
ALT: Alanine aminotransferase
AST: Aspartate transaminase
GLU: Glucose
FBG: The fasting blood-glucose
H&E: Hematoxylin and eosin
IHC: Immunohistochemical
Col-1: Collagen type 1
α-SMA: α-Smooth muscle actin
KCs: Kupffer cells
DCs: Dendritic cells
TNF-α: Tumor necrosis factor-α
IFN-γ: γ-Interferon
NK cells: Natural killer
NKT: Natural killer T cells
MPO: Myeloperoxidase
CCL: C–C motif chemokine
CXCL: C-X-C motif chemokine
G-CSF: Macrophage colony stimulating factor
GM-CSF: Granulocyte–macrophage colony stimulating factor

## References

[1] Brunt EM, Wong VW, Nobili V, Day CP, Sookoian S, Maher JJ (2015). Nonalcoholic fatty liver disease. Nat Rev Dis Primers.

[2] Basaranoglu M, Basaranoglu G, Bugianesi E (2015). Carbohydrate intake and nonalcoholic fatty liver disease: fructose as a weapon of mass destruction. Hepatobiliary Surg Nutr.

[3] Kohli R, Kirby M, Xanthakos SA, Softic S, Feldstein AE, Saxena V (2010). High-fructose, medium chain trans fat diet induces liver fibrosis and elevates plasma coenzyme Q9 in a novel murine model of obesity and nonalcoholic steatohepatitis. Hepatology.

[4] Obara N, Fukushima K, Ueno Y, Wakui Y, Kimura O, Tamai K (2010). Possible involvement and the mechanisms of excess trans-fatty acid consumption in severe NAFLD in mice. J Hepatol.

[5] Trevaskis JL, Griffin PS, Wittmer C, Neuschwander-Tetri BA, Brunt EM, Dolman CS (2012). Glucagon-like peptide-1 receptor agonism improves metabolic, biochemical, and histopathological indices of nonalcoholic steatohepatitis in mice. Am J Physiol Gastrointest Liver Physiol.

[6] Eng JM, Estall JL. Diet-induced models of non-alcoholic fatty liver disease: food for thought on sugar, fat, and cholesterol. Cells 2021; 10(7). 10.3390/cells10071805 PMC8303413 34359974

[7] Kim B, Farruggia C, Ku CS, Pham TX, Yang Y, Bae M (2017). Astaxanthin inhibits inflammation and fibrosis in the liver and adipose tissue of mouse models of diet-induced obesity and nonalcoholic steatohepatitis. J Nutr Biochem.

[8] Narayanan S, Surette FA, Hahn YS (2016). the immune landscape in nonalcoholic steatohepatitis. Immune Netw.

[9] Hoogerland JA, Staels B, Dombrowicz D (2022). Immune-metabolic interactions in homeostasis and the progression to NASH. Trends Endocrinol Metab.

[10] Bedossa P, Consortium FP (2014). Utility and appropriateness of the fatty liver inhibition of progression (FLIP) algorithm and steatosis, activity, and fibrosis (SAF) score in the evaluation of biopsies of nonalcoholic fatty liver disease. Hepatology.

[11] Sheka AC, Adeyi O, Thompson J, Hameed B, Crawford PA, Ikramuddin S (2020). nonalcoholic steatohepatitis: a review. JAMA.

[12] Im YR, Hunter H, de Gracia HD, Duret A, Cheah Q, Dong J (2021). A systematic review of animal models of NAFLD finds high-fat, high-fructose diets most closely resemble human NAFLD. Hepatology.

[13] Linden MA, Sheldon RD, Meers GM, Ortinau LC, Morris EM, Booth FW (2016). Aerobic exercise training in the treatment of non-alcoholic fatty liver disease related fibrosis. J Physiol.

[14] Ito M, Suzuki J, Tsujioka S, Sasaki M, Gomori A, Shirakura T (2007). Longitudinal analysis of murine steatohepatitis model induced by chronic exposure to high-fat diet. Hepatol Res.

[15] Mells JE, Fu PP, Kumar P, Smith T, Karpen SJ, Anania FA (2015). Saturated fat and cholesterol are critical to inducing murine metabolic syndrome with robust nonalcoholic steatohepatitis. J Nutr Biochem.

[16] Tu LN, Showalter MR, Cajka T, Fan S, Pillai VV, Fiehn O (2017). Metabolomic characteristics of cholesterol-induced non-obese nonalcoholic fatty liver disease in mice. Sci Rep.

[17] Watanabe M, Morimoto K, Houten SM, Kaneko-Iwasaki N, Sugizaki T, Horai Y (2012). Bile acid binding resin improves metabolic control through the induction of energy expenditure. PLoS ONE.

[18] Huby T, Gautier EL (2022). Immune cell-mediated features of non-alcoholic steatohepatitis. Nat Rev Immunol.

[19] Lalor PF, Faint J, Aarbodem Y, Hubscher SG, Adams DH (2007). The role of cytokines and chemokines in the development of steatohepatitis. Semin Liver Dis.

[20] Gadd VL, Skoien R, Powell EE, Fagan KJ, Winterford C, Horsfall L (2014). The portal inflammatory infiltrate and ductular reaction in human nonalcoholic fatty liver disease. Hepatology.

[21] Syn WK, Agboola KM, Swiderska M, Michelotti GA, Liaskou E, Pang H (2012). NKT-associated hedgehog and osteopontin drive fibrogenesis in non-alcoholic fatty liver disease. Gut.

[22] Kiki I, Altunkaynak BZ, Altunkaynak ME, Vuraler O, Unal D, Kaplan S (2007). Effect of high fat diet on the volume of liver and quantitative feature of Kupffer cells in the female rat: a stereological and ultrastructural study. Obes Surg.

[23] Dywicki J, Buitrago-Molina LE, Noyan F, Davalos-Misslitz AC, Hupa-Breier KL, Lieber M (2022). The detrimental role of regulatory T cells in nonalcoholic steatohepatitis. Hepatol Commun.

[24] Diehl AM (2002). Nonalcoholic steatosis and steatohepatitis IV. Nonalcoholic fatty liver disease abnormalities in macrophage function and cytokines. Am J Physiol Gastrointest Liver Physiol.

[25] Connolly MK, Bedrosian AS, Mallen-St Clair J, Mitchell AP, Ibrahim J, Stroud A (2009). In liver fibrosis, dendritic cells govern hepatic inflammation in mice via TNF-alpha. J Clin Invest.

[26] Wang F, Zhang X, Liu W, Zhou Y, Wei W, Liu D (2022). Activated natural killer cell promotes nonalcoholic steatohepatitis through mediating JAK/STAT pathway. Cell Mol Gastroenterol Hepatol.

[27] Sutti S, Jindal A, Locatelli I, Vacchiano M, Gigliotti L, Bozzola C (2014). Adaptive immune responses triggered by oxidative stress contribute to hepatic inflammation in NASH. Hepatology.

[28] Ou R, Liu J, Lv M, Wang J, Wang J, Zhu L (2017). Neutrophil depletion improves diet-induced non-alcoholic fatty liver disease in mice. Endocrine.

[29] Stefanovic-Racic M, Yang X, Turner MS, Mantell BS, Stolz DB, Sumpter TL (2012). Dendritic cells promote macrophage infiltration and comprise a substantial proportion of obesity-associated increases in CD11c+ cells in adipose tissue and liver. Diabetes.

[30] Sbierski-Kind J, Kath J, Brachs S, Streitz M, von Herrath MG, Kuhl AA (2018). Distinct housing conditions reveal a major impact of adaptive immunity on the course of obesity-induced Type 2 diabetes. Front Immunol.

[31] Zheng S, Yang W, Yao D, Tang S, Hou J, Chang X (2022). A comparative study on roles of natural killer T cells in two diet-induced non-alcoholic steatohepatitis-related fibrosis in mice. Ann Med.

[32] Heymann F, Tacke F (2016). Immunology in the liver–from homeostasis to disease. Nat Rev Gastroenterol Hepatol.

