# Supplementary figures and images for: A high-trans fat, high-carbohydrate, high-cholesterol, high-cholate diet-induced nonalcoholic steatohepatitis mouse model and its hepatic immune response
Source: Nutr Metab (Lond). 2023 May 27;20:28. doi: 10.1186/s12986-023-00749-w (PMC10224300; doi:10.1186/s12986-023-00749-w)

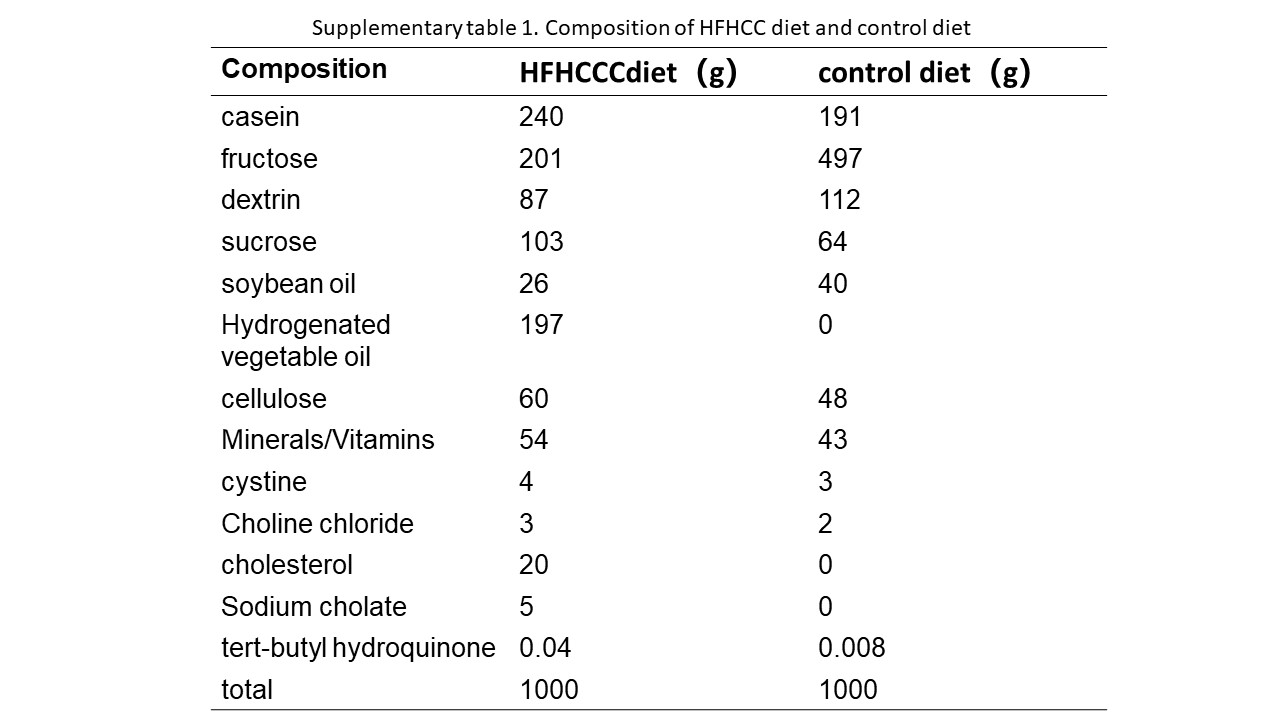

Supplement: Supplementary file 1 — Additional file 1. Composition of HFHCC diet and control diet. [file 12986_2023_749_MOESM1_ESM.jpg]

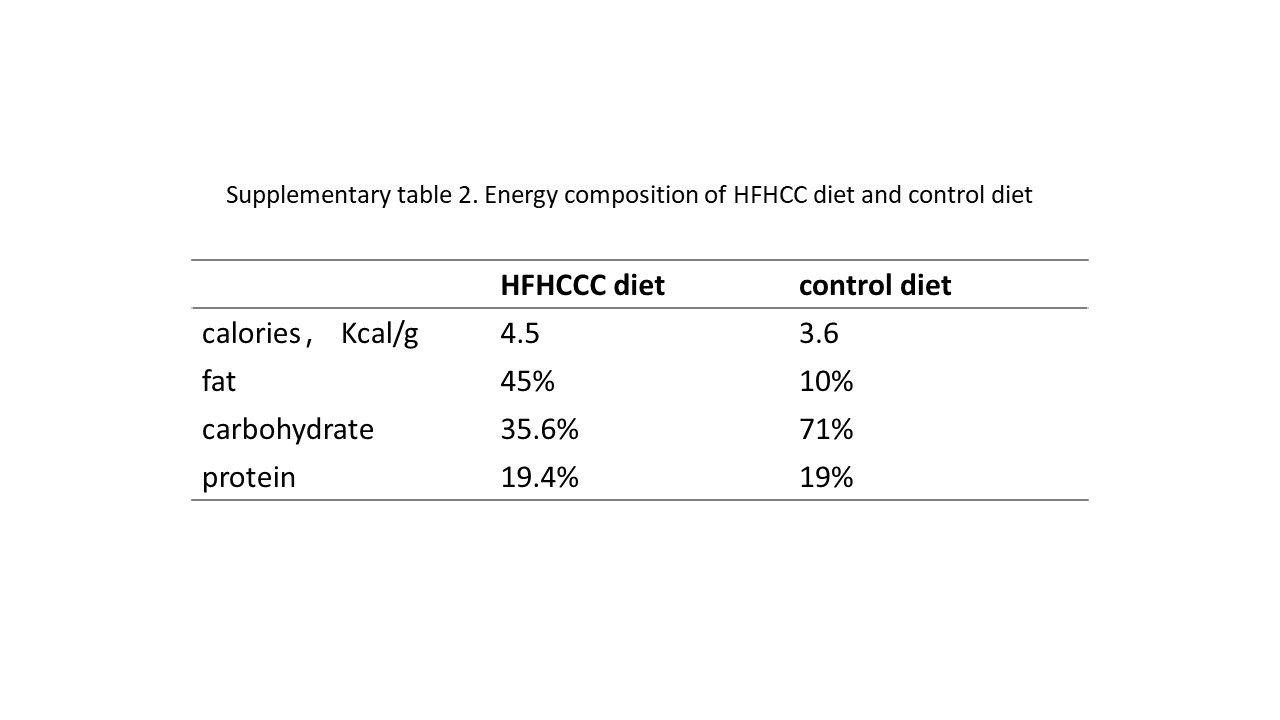

Supplement: Supplementary file 2 — Additional file 2. Energy composition of HFHCC diet and control diet. [file 12986_2023_749_MOESM2_ESM.jpg]
